# The prevalence of non-contrast CT imaging abnormalities in reversible cerebral vasoconstriction syndrome: A systematic review and meta-analysis

**DOI:** 10.1371/journal.pone.0295558

**Published:** 2024-03-11

**Authors:** Ryan Daniel Gotesman, Naomi Niznick, Brian Dewar, Dean A. Fergusson, Ranjeeta Mallick, Risa Shorr, Michel Shamy, Dar Dowlatshahi

**Affiliations:** 1 Department of Family Medicine, McMaster University, Kitchener, Canada; 2 Department of Medicine (Critical Care), The Ottawa Hospital, Ottawa, Canada; 3 Ottawa Hospital Research Institute, Ottawa, Canada; 4 Department of Medicine, University of Ottawa, Ottawa, Canada; 5 Faculty of Medicine, University of Ottawa, Ottawa, Canada; 6 The Ottawa Hospital, Ottawa, Canada; Bach Mai Hospital, VIET NAM

## Abstract

**Background:**

Reversible cerebral vasoconstriction syndrome (RCVS) is a syndrome of recurrent thunderclap headaches and reversible vasoconstriction of the cerebral arteries on neuroimaging within 3 months of onset. Initial non-contrast computed tomography (CT) can reveal abnormalities such as ischemic stroke, intracerebral hemorrhage, and subarachnoid hemorrhage (SAH) can be present in patients with RCVS and may delay diagnosis.

**Aims:**

We conducted a systematic review and meta-analysis in accordance with the PRISMA guidelines. We aimed to estimate the prevalence of imaging abnormalities on initial non-contrast CT head in adult patients with RCVS.

**Data sources & eligibility criteria:**

We searched electronic databases including MEDLINE, EMBASE, and the Cochrane Register of Clinical Trials from inception to August 2, 2022. Eligible studies included articles reporting the prevalence of non-contrast CT abnormalities on initial neuroimaging in patients with RCVS, aged 18 and older. Case series, observational studies and clinical trials were included. Data was extracted directly from included papers using a standardized data charting form.

**Results:**

The search yielded 722 titles with duplicates removed. Twenty studies that included 379 patients with RCVS met inclusion criteria. We classified non-contrast CT abnormalities as either ischemic stroke, ICH, or SAH. We pooled prevalence data using a random effects model with the inverse-variance weighted method. The most common imaging finding was SAH with a pooled prevalence of 24% (95% CI:17%-33%), followed by ICH at 14% (95% CI:8%-22%), and ischemic stroke at 10% (95% CI:7%-14%). The pooled prevalence of any of these imaging abnormalities on initial non-contrast CT was 31% (95% CI:23%-40%). Risk of bias was moderate to very-high-risk for case-series and low-risk for observational studies.

**Conclusion:**

Our review demonstrates that one-third of patients with RCVS will have an abnormality on initial non-contrast CT head, including either an ischemic stroke, ICH, or SAH. These findings highlight the diagnostic challenges of RCVS imaging and contribute to our understanding of this disease.

## Introduction

Reversible cerebral vasoconstriction syndrome (RCVS) is a syndrome of recurrent thunderclap headaches and segmental vasoconstriction of cerebral arteries on neuroimaging, which is reversible within 3 months of onset [1–3]. Accompanying neurologic symptoms, such as focal deficits or seizures, may occur. The diagnosis of RCVS is based on clinical symptoms and characteristic angiographic findings on cerebral vessels imaging [3–5]. Various diagnostic criteria have been proposed, including the International Classification of Headache Disorders criteria and the RCVS_2_ score, however the diagnosis of RCVS poses significant challenges due to its variable clinical presentation and overlapping features with other conditions such as primary angiitis of the central nervous system (PACNS) and posterior reversible encephalopathy syndrome (PRES) [2,3,5].

Non-contrast computed tomography (CT) of the head is often the first imaging modality acquired and is useful for screening for other intracranial pathology requiring acute specific treatments, such as aneurysmal subarachnoid hemorrhage (SAH), acute ischemic stroke, and spontaneous intracranial hemorrhage (ICH) [6,7]. CT imaging is used in conjunction with cerebral vessel imaging, such as computed tomography angiography (CTA) or magnetic resonance angiography (MRA), to assess the cerebral vasculature [8,9]. Early imaging in RCVS is often normal, after which patients classically experience recurrent thunderclap headache prompting a return to medical care for repeat imaging. Imaging abnormalities can be detected on delayed or repeat imaging, with the characteristic cerebral vasoconstriction reaching its peak after 2–3 weeks of symptom onset. (2) Digital subtraction angiography (DSA) can also be performed when suspicion of RCVS is high, and non-invasive imaging has been unremarkable [8]. Multifocal segmental narrowing and dilatation of the cerebral arteries is the classic angiographic finding in RCVS [6,10].

RCVS can be complicated by ischemic stroke, ICH and SAH [6,10]. The co-existence of these findings on initial imaging can contribute to diagnostic uncertainty, with the initial differential diagnosis often including vasculitis, aneurysmal SAH and cerebral venous sinus thrombosis [11]. The frequency of initial abnormal non-contrast CT head findings in RCVS varies widely in the published literature with estimates for ischemic stroke prevalence ranging from 8–39%, SAH from 15–34% and ICH from 6–20% [12–16]. Uncertainty on the basis of imaging findings can delay diagnosis, even when the clinical history is strongly suggestive of RCVS, leading to delays in disease-specific management.

In this study, we aimed to establish the frequency of imaging abnormalities on baseline non-contrast CT head in patients with RCVS. We conducted a systematic review and meta-analysis to estimate the prevalence of abnormal findings consistent with ischemic stroke, ICH and SAH on non-contrast CT in adult patients with RCVS.

## Methods

We conducted a systematic review and meta-analysis following the Preferred Reporting Items for Systematic Reviews and Meta-Analyses (PRISMA) reporting guidelines [17]. A detailed protocol of the study design and methods was registered (registration number CRD42020190637) and published a priori [18].

### Study search

We performed a search of Medline, Embase (Embase Classic + Embase), and the Cochrane library from inception to August 2, 2022. We developed a structured search strategy in consultation with a health science librarian using controlled vocabulary and relevant key terms. Reference lists of included studies were also reviewed for potential inclusion. The complete search strategies are available in the Supplementary Materials, (S1 Table).

### Eligibility criteria and study selection

Research studies were selected for inclusions if they reported the prevalence of neuroimaging abnormalities on initial non-contrast CT in patients with RCVS, diagnosed based on characteristic clinical and imaging criteria using CTA, MRA or DSA. Eligible studies included case-series, observational studies, and clinical trials with adult patients 18 years or older, published in English. Case reports, abstracts and commentaries were excluded, in addition to studies reporting initial MRI rather than CT findings.

We used Covidence (Covidence, Melbourne) to screen citations for inclusion. Citations were screened independently by at least two trained reviewers (RDG, NN) at the title, abstract and full-text level [19]. Reviewers met to resolve discrepancies after 20% of titles and abstracts had been reviewed. Conflicts were resolved by consensus. Full texts of included studies were retrieved, and data was extracted by two independent reviewers (RDG, NN) onto standardized data reporting forms.

### Risk of bias assessment

The methodological quality of case series was assessed using the Institute of Health Economics Quality Appraisal Checklist for Case Series Studies [20]. The checklist is comprised of 20 criteria that can be answered with ‘yes’ or ‘no’ and then used to measure the quality of a study. Two criteria were not considered as they were deemed not applicable to our study. Low risk of bias was defined as 0–2 ‘no’ responses, moderate risk as 3–5 ‘no’ responses, high risk as 6–8 ‘no’ responses and > = 9 ‘no’ responses defining very high risk of bias studies. The quality of observational studies was assessed using the Newcastle-Ottawa Scale for assessing the quality of nonrandomised studies in meta-analyses [21]. Low risk of bias using this scale is defined as a score between 6–9, moderate as between 4–5 and high as between 1–3.

### Statistical analysis

The prevalence of acute ischemic stroke, ICH or SAH on non-contrast CT was calculated by taking the number of individuals with RCVS and one of these abnormalities and dividing by the total number of patients with RCVS in a study. Prevalence estimates were pooled using a random effects model with inverse-variance weighting in order to account for heterogeneity among studies and put more weight on studies with higher precision. A continuity correction was used to account for studies where prevalence was 0. Heterogeneity was estimated with the I^2^ statistic. An I^2^ between 0–35% was considered low heterogeneity, 35–55% as moderate heterogeneity, 55–83% as substantial heterogeneity and 83%-100% as considerable heterogeneity [22]. Data was visually depicted using Forest plots. Analysis was completed with Comprehensive Meta Analysis v2.2.064 (Biostat Inc, NJ, USA).

## Results

**Fig 1:**
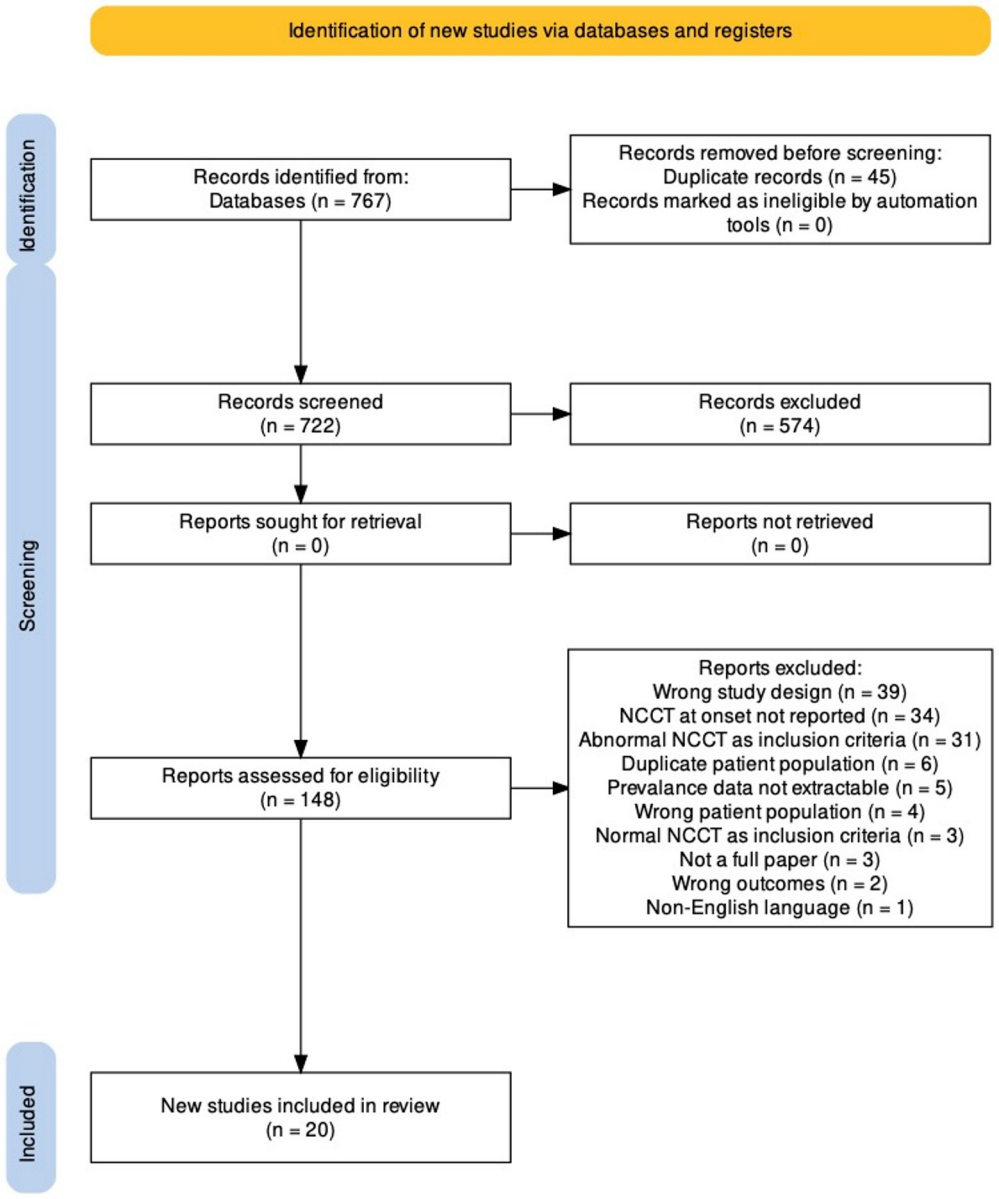
Preferred reporting items for systematic reviews and meta analyses flow diagram.

Our electronic database searched yielded 767 studies. With duplicates removed, 722 titles and abstracts were screened, and 148 full-text articles were subsequently reviewed. No additional publications were included after reviewing the reference lists. Twenty articles met inclusion criteria and were included in the meta-analysis (Fig 1; S2 Table).

There was a total of 379 patients with RCVS across the 20 studies (77 men and 302 women). The study-level pooled mean (SD) age of patients with RCVS was 45.5 (7.7) years. The imaging modalities used to diagnose RCVS were CT/CTA, MRI/MRA, DSA and transcranial doppler; CT/CTA was the most common. With respect to study design, 16 studies were case series and the remaining 4 were observational studies. Seven studies (35%) included criteria used to diagnose RCVS based on imaging and 6 studies (30%) included clinical criteria used to diagnose RCVS.

**Fig 2:**
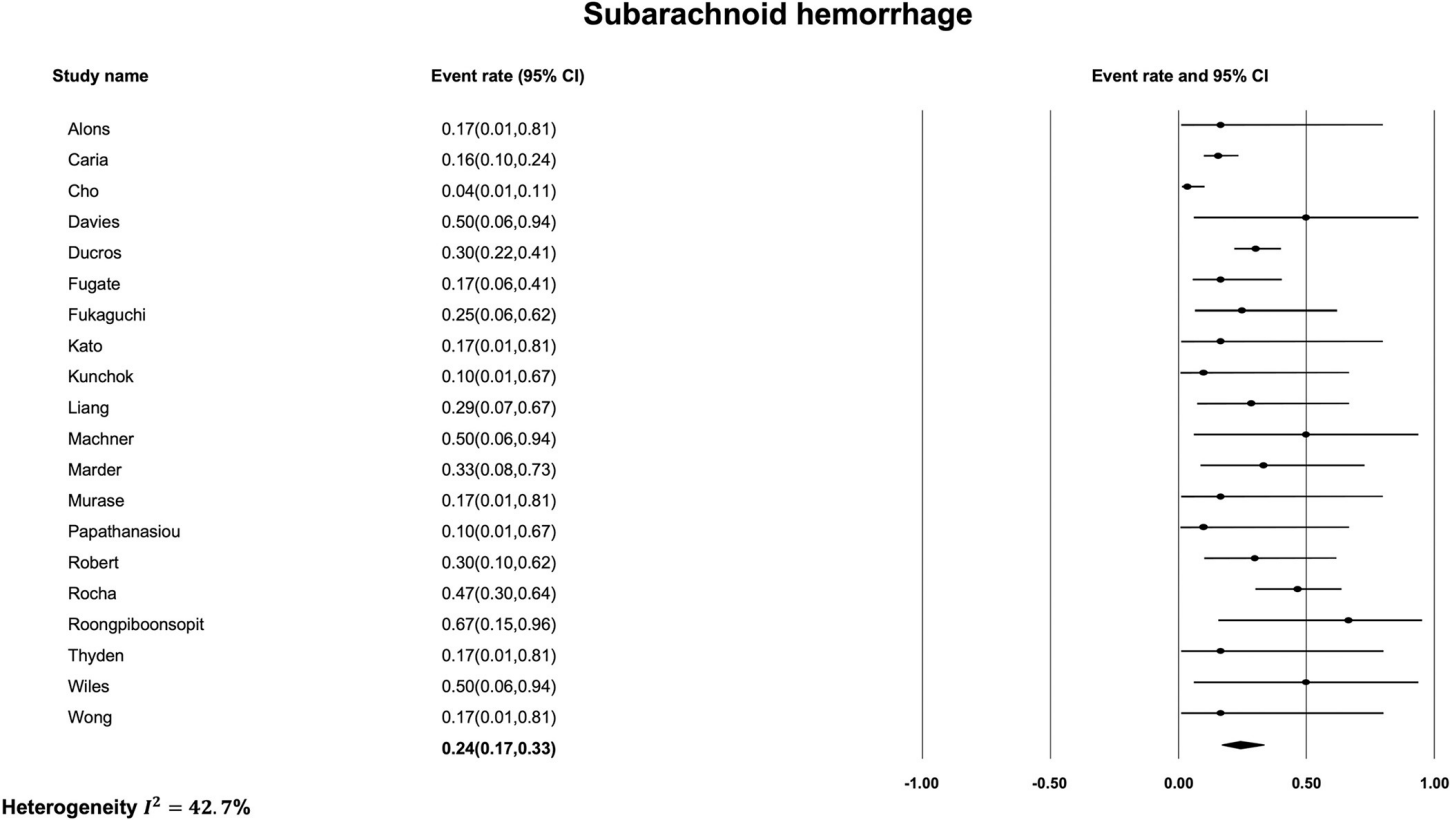
Prevalence of subarachnoid hemorrhage on initial non-contrast CT.

**Fig 3:**
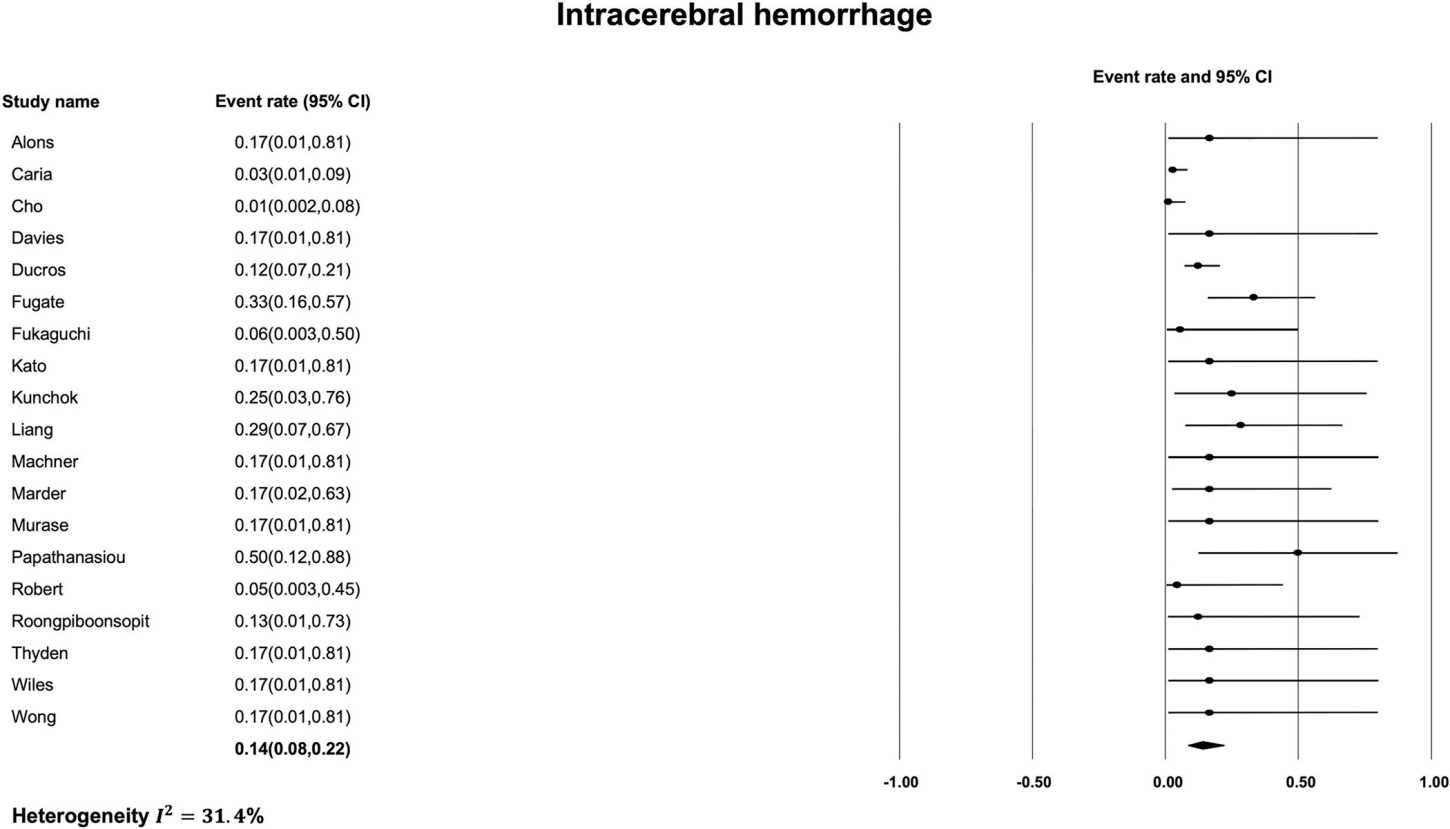
Prevalence of intracerebral hemorrhage on initial non-contrast CT.

**Fig 4:**
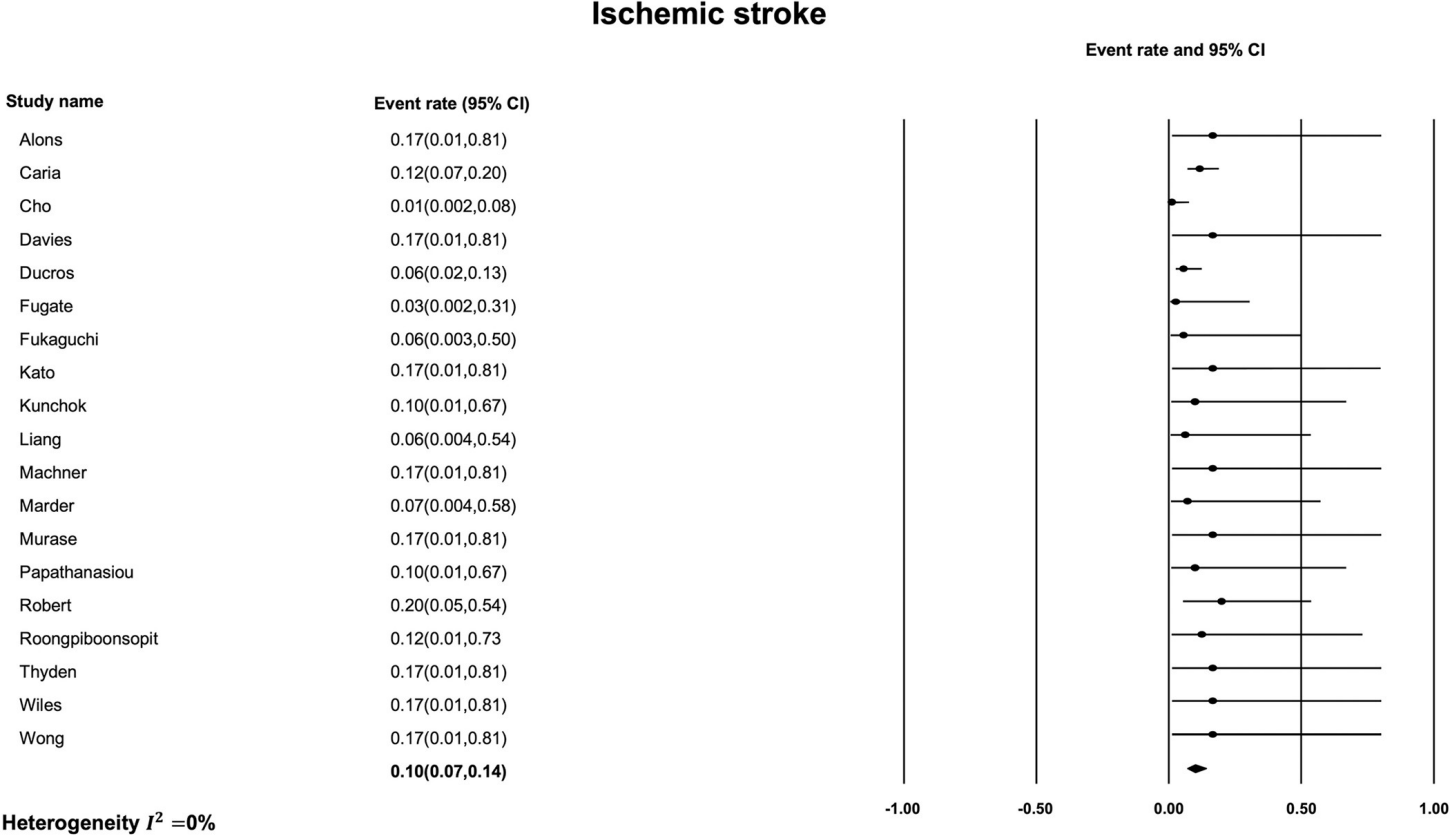
Prevalence of ischemic stroke on initial non-contrast CT.

**Fig 5:**
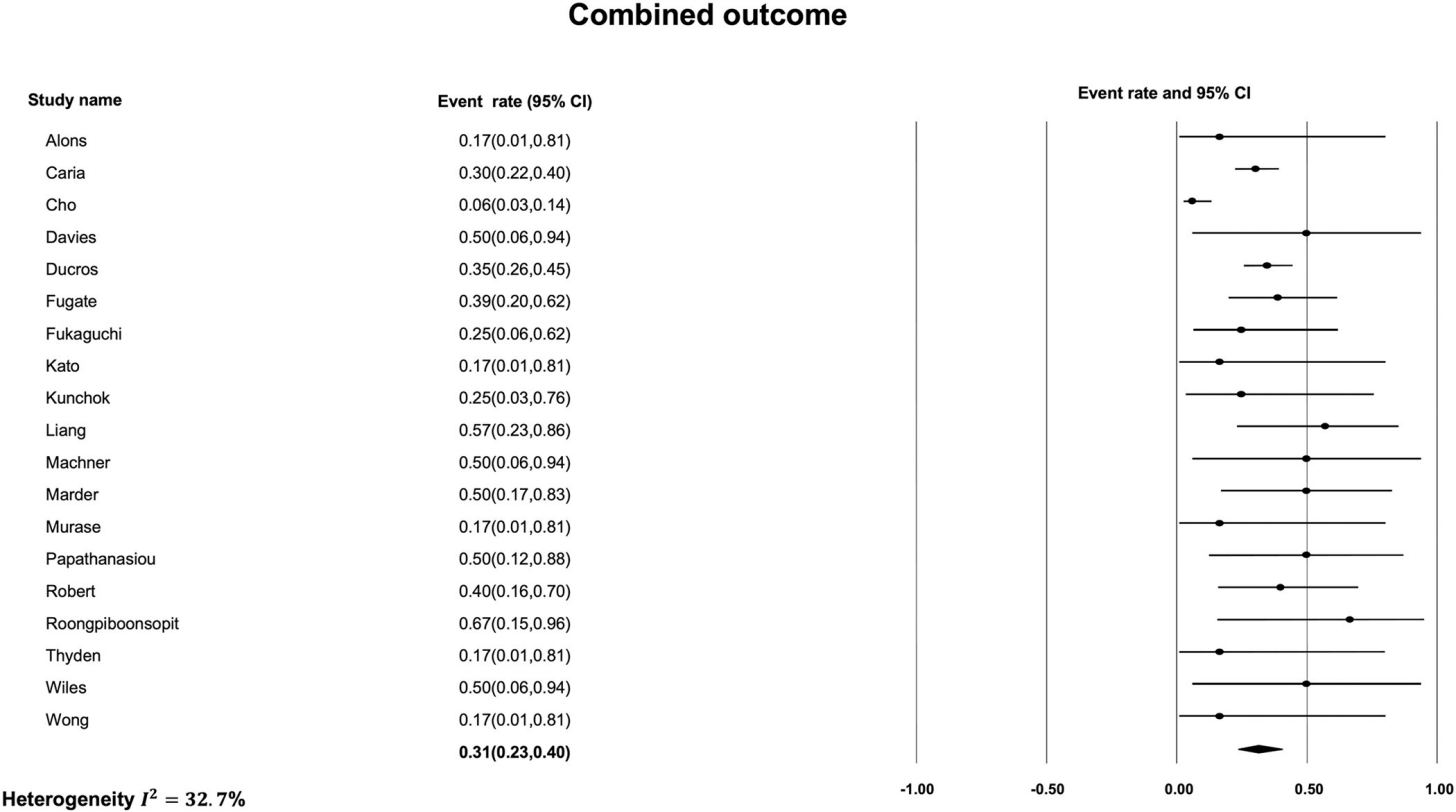
Prevalence of either ischemic stroke, intracerebral hemorrhage, or subarachnoid hemorrhage on initial non-contrast CT.

The most common finding on initial non-contrast CT was subarachnoid hemorrhage (Fig 2) with a pooled prevalence of 24% [95% CI, 17% to 33%]. The estimated I^2^ was 42.7% indicating moderate heterogeneity between studies. The next most common imaging finding on initial non-contrast CT in patients with RCVS was intracerebral hemorrhage (Fig 3) with a pooled prevalence of 14% [95% CI, 8% to 22%]. The estimated I^2^ was 31.4% indicating low heterogeneity. The least frequent non-contrast CT imaging finding was ischemic stroke (Fig 4) with a pooled prevalence of 10% [95% CI, 7% to 14%]. The estimated I^2^ was 0%, indicating low heterogeneity. Finally, the pooled prevalence of any of ischemic stroke, ICH or SAH, on the initial non-contrast CT of a patient with RCVS was 31% [95% CI, 23% to 40%] (Fig 5) with an estimated I^2^ of 32.7% indicating low heterogeneity.

Assessment of risk of bias (S3 Table) for case series studies revealed 2 studies with moderate risk of bias, 3 with high risk of bias and 11 with very high risk of bias. The 4 observational studies were all found to have low risk of bias.

## Discussion

This meta-analysis provides prevalence estimates for commonly encountered imaging abnormalities on initial non-contrast CT head in patients with RCVS. While it is known that patients with RCVS can have abnormalities on baseline neuroimaging, the frequency of these findings has previously been unclear [6,16,23]. Our synthesis and meta-analysis of the published data reveals an imaging finding prevalence of 24%, 14% and 10% for SAH, ICH and ischemic stroke, respectively, with 31% of patients having any of these findings on baseline CT head.

The varied presentation of RCVS along with the overlapping imaging findings with other medical conditions poses challenges in achieving a timely and accurate diagnosis. Early recognition of RCVS is crucial due to its potential for significant morbidity, the need for specific management strategies, in order to decrease the risk of complications, and to avoid inappropriate therapies associated with misdiagnosis [24]. While there exist consensus clinical and neuroimaging diagnostic criteria, it remains challenging to accurately diagnose RCVS especially when there abnormalities are present on a patient’s initial non-contrast CT head, such as ischemic stroke, ICH, and SAH [1,2,5,13]. These findings can lead clinicians to alternative diagnosis such as PACNS, aSAH or PRES [12,25]. This may result in unnecessary investigations, inappropriate interventions, for example the initiation of corticosteroids for possible PACNS, and suboptimal outcomes [12,16,23]. Our study provides reassurance that these abnormalities are common in RCVS, with approximately one third of patients having either an ischemic stroke, ICH or SAH on their baseline CT head. The presence of these findings should not dissuade a clinician from diagnosing RCVS when the clinical history, presentation, and vessel imaging are consistent with RCVS. In particular, clinicians should take into the account the time course of clinical symptoms and imaging findings: imaging can be normal early in the clinical course and “baseline” CT may be obtained only after recurrence of thunderclap headaches with the key diagnostic features of vasospasm peaking at 2–3 weeks after symptom onset.

The strengths of our meta-analysis are that we prospectively registered our study, employed a detailed protocol, and systematically searched the literature. Our meta-analysis was also strengthened by the low heterogeneity for ischemic stroke, ICH and combined outcomes, and a moderate heterogeneity for SAH. Our study is not without limitations. Sixteen of the included studies were case series with moderate to very high risk of bias. This is most likely due to publication bias for more severe case presentations with a bias towards abnormal imaging. However, these 16 studies contributed only 46 (12%) patients and the remaining 333 patients were from cohort studies with low risk of bias. The overall prevalence estimates in stroke, ICH and SAH are therefore driven primarily by the cohort studies. Due to the nature of the available data, we were not able assess for effect of sex, age, ethnicity, or race on the prevalence of imaging findings. An independent patient data meta-analysis level will be required to assess these important exposure variables. Finally, RCVS can have a variable clinical presentation and potentially overlap with related conditions such as posterior reversible encephalopathy syndromes. Moreover, the presenting features of RCVS may differ in those diagnosed in emergency departments versus those in ambulatory care clinics. These factors may have introduced additional bias into the meta-analysis.

In summary, our meta-analysis demonstrates that one-third of patients with RCVS will have either an ischemic stroke, ICH, or SAH, on initial non-contrast CT head. Our study highlights that non-contrast CT head abnormalities are common in RCVS and their presence should not delay diagnosis and management.

## Supporting information

S1 Checklist. PRISMA 2020 checklist (PDF)

S1 Table Search strategy. (DOCX)

S2 Table List of included studies. (DOCX)

S3 Table Risk of bias using the institute of health economics quality appraisal checklist for case series studies and the Newcastle-Ottawa Scale for assessing the quality of non-randomized studies in meta-analyses. (DOCX)
