# Supplementary material for: The prevalence of non-contrast CT imaging abnormalities in reversible cerebral vasoconstriction syndrome: A systematic review and meta-analysis
Source: PLoS One. 2024 Mar 11;19(3):e0295558. doi: 10.1371/journal.pone.0295558 (PMC10927111; doi:10.1371/journal.pone.0295558)
Supplement: S1 Table — (DOCX) [file pone.0295558.s002.docx]

**Supplemental Material**

**S1 Table:** Search strategy

The MEDLINE, EMBASE, and Cochrane Central Register on Controlled Trials databases were searched on August 2, 2022, using the following search strategy:

1. Vasospasm, Intracranial/ or Headache Disorders, Primary/ (10841)
2. Vasoconstriction/ and exp Cerebrovascular Disorders/ (3047)
3. ((intracranial or intracereb* or intercereb* or cerebrum or cerebral or brain) and (vasoconstrict* or vasospasm*)).tw,kf. (27084)
4. or/1-3 (33053)
5. reversible.tw,kw. (331842)
6. 4 and 5 (2251)
7. rcvs.tw,kw. (1936)
8. (Call-Fleming or benign angiopathy of the central nervous system or acute benign cerebral angiopathy or postpartum angiopathy or thunderclap headache with reversible vasospasm or migrainous vasospasm or migraine angiitis or drug-induced cerebral arteritis or drug-induced arteritis or drug-unded cerebral angiopathy or drug-induced angiopathy or CNS pseudovasculitis).tw,kw. (211)
9. or/6-8 (3479)
10. tomography, x-ray computed/ or computed tomography angiography/ (458137)
11. (ct or computed tomograph*).tw,kw. (1290654)
12. angiography/ or angiography, digital subtraction/ or cerebral angiography/ (251469)
13. (angiograph* or angiogram*).tw,kw. (516573)
14. magnetic resonance imaging/ or magnetic resonance angiography/ (966147)
15. (mri or magnetic resonance imag*).tw,kw. (1000047)
16. Ultrasonography, Doppler, Transcranial/ (9652)
17. transcran* doppler*.tw,kw. or (transcran* ultrasonograph* or transcran* sonograph* or transcran* ultrasound*).kw. (23105)
18. (transcran* adj2 (ultrasonograph* or sonograph* or ultrasound*)).tw. (12072)
19. (neuroimag* or neuro imag*).tw,kw. (131450)
20. or/10-19 (3168483)
21. 9 and 20 (1694)
22. exp brain ischemia/ or exp stroke/ (549189)
23. stroke*.tw,kw. (702880)
24. ((brain or cerebral) adj3 isch?em*).tw. (115810)
25. ((brain or cerebral) and isch?em*).kf. (6322)
26. Brain Edema/ or Edema/ (207804)
27. ed?ema*.tw,kw. (310397)
28. intracranial hemorrhages/ or exp subarachnoid hemorrhage/ (123665)
29. h?emorrhag*.tw,kw. (674036)
30. sah.tw,kw. (26870)
31. (isch?em* adj2 infarct*).tw. (20021)
32. isch?em* infarct*.kw. (181)
33. or/22-32 (1923029)
34. 21 and 33 (1386)
35. exp animals/ not humans/ (17829136)
36. 34 not 35 (864)
37. limit 36 to english language (794)
38. **37** **use** **medall** **(338)** **Medline**
39. reversible cerebral vasoconstriction syndrome/ (976)
40. ((intracranial or intracereb* or intercereb* or cerebrum or cerebral or brain) and (vasoconstrict* or vasospasm*) and reversible).tw. (2142)
41. rcvs.tw. (1917)
42. (Call-Fleming or benign angiopathy of the central nervous system or acute benign cerebral angiopathy or postpartum angiopathy or thunderclap headache with reversible vasospasm or migrainous vasospasm or migraine angiitis or drug-induced cerebral arteritis or drug-induced arteritis or drug-unded cerebral angiopathy or drug-induced angiopathy or CNS pseudovasculitis).tw. (188)
43. or/39-42 (3549)
44. *computer assisted tomography/ or computed tomographic angiography/ (175821)
45. (ct or computed tomograph*).tw. (1268996)
46. *angiography/ or exp brain angiography/ or *digital subtraction angiography/ (85232)
47. (angiograph* or angiogram*).tw. (502770)
48. magnetic resonance angiography/ or *nuclear magnetic resonance imaging/ (229626)
49. (mri or magnetic resonance imag*).tw. (956815)
50. transcranial doppler ultrasonography/ (9223)
51. (transcran* adj2 (ultrasonograph* or sonograph* or ultrasound* or doppler)).tw. (24783)
52. *neuroimaging/ or *functional neuroimaging/ (30347)
53. (neuroimag* or neuro imag*).tw. (121906)
54. or/44-53 (2625546)
55. 43 and 54 (1606)
56. cerebrovascular accident/ (305682)
57. exp brain ischemia/ (296962)
58. stroke*.tw. (687694)
59. ((brain or cerebral) adj3 isch?em*).tw. (115810)
60. brain edema/ (49139)
61. ed?ema*.tw. (301725)
62. subarachnoid hemorrhage/ (66623)
63. brain hemorrhage/ (112308)
64. h?emorrhag*.tw. (651322)
65. sah.tw. (26518)
66. (isch?em* adj2 infarct*).tw. (20021)
67. or/56-66 (1812092)
68. 55 and 67 (1343)
69. (exp animals/ or animal experiment/) not exp humans/ (10414540)
70. 68 not 69 (1326)
71. limit 70 to english language (1234)
72. conference abstract.pt. (3784455)
73. 71 not 72 (883)
74. **73** **use** **emczd** **(588)** **Embase**
75. Vasospasm, Intracranial/ or Headache Disorders, Primary/ (10841)
76. Vasoconstriction/ and exp Cerebrovascular Disorders/ (3047)
77. ((intracranial or intracereb* or intercereb* or cerebrum or cerebral or brain) and (vasoconstrict* or vasospasm*)).tw,kw. (27730)
78. or/75-77 (33585)
79. reversible.tw,kw. (331842)
80. 78 and 79 (2273)
81. rcvs.tw,kw. (1936)
82. (Call-Fleming or benign angiopathy of the central nervous system or acute benign cerebral angiopathy or postpartum angiopathy or thunderclap headache with reversible vasospasm or migrainous vasospasm or migraine angiitis or drug-induced cerebral arteritis or drug-induced arteritis or drug-unded cerebral angiopathy or drug-induced angiopathy or CNS pseudovasculitis).tw,kw. (211)
83. or/80-82 (3494)
84. tomography, x-ray computed/ or computed tomography angiography/ (458137)
85. (ct or computed tomograph*).tw,kw. (1290654)
86. angiography/ or angiography, digital subtraction/ or cerebral angiography/ (251469)
87. (angiograph* or angiogram*).tw,kw. (516573)
88. magnetic resonance imaging/ or magnetic resonance angiography/ (966147)
89. (mri or magnetic resonance imag*).tw,kw. (1000047)
90. Ultrasonography, Doppler, Transcranial/ (9652)
91. transcran* doppler*.tw,kw. or (transcran* ultrasonograph* or transcran* sonograph* or transcran* ultrasound*).kw. (23105)
92. (transcran* adj2 (ultrasonograph* or sonograph* or ultrasound*)).tw. (12072)
93. (neuroimag* or neuro imag*).tw,kw. (131450)
94. or/84-93 (3168483)
95. 83 and 94 (1708)
96. exp brain ischemia/ or exp stroke/ (549189)
97. stroke*.tw,kw. (702880)
98. ((brain or cerebral) adj3 isch?em*).tw. (115810)
99. (brain isch?em* or cerebral isch?em*).kw. (22194)
100. Brain Edema/ or Edema/ (207804)
101. ed?ema*.tw,kw. (310397)
102. intracranial hemorrhages/ or exp subarachnoid hemorrhage/ (123665)
103. h?emorrhag*.tw,kw. (674036)
104. sah.tw,kw. (26870)
105. (isch?em* adj2 infarct*).tw. (20021)
106. isch?em* infarct*.kw. (181)
107. or/96-106 (1923351)
108. 95 and 107 (1398)
109. limit 108 to english language (1297)
110. **109** **use** **cctr** **(5)** **Cochrane**
111. 38 or 74 or 110 (931)
112. remove duplicates from 111 (644)
113. **112** **use** **medall** **(337)**
114. **112** **use** **emczd** **(303)**
115. **112** **use** **cctr** **(4)**
