# Supplementary material for: The prevalence of non-contrast CT imaging abnormalities in reversible cerebral vasoconstriction syndrome: A systematic review and meta-analysis
Source: PLoS One. 2024 Mar 11;19(3):e0295558. doi: 10.1371/journal.pone.0295558 (PMC10927111; doi:10.1371/journal.pone.0295558)
Supplement: S2 Table — (DOCX) [file pone.0295558.s003.docx]

**Supplemental Material**

**S2 Table:** List of included studies

| Author Last Name | Article Title | Journal Title | Publication year | Study Design |
| --- | --- | --- | --- | --- |
| Davies | The reversible cerebral vasoconstriction syndrome in association with venlafaxine and methenamine | BMJ Case Reports | 2013 | Case series |
| Fukaguchi | Reversible cerebral vasoconstriction syndrome: the importance of follow-up imaging within 2 weeks. | Acute Medicine & Surgery | 2020 | Case series |
| Kato | Triptan-induced reversible cerebral vasoconstriction syndrome: Two case reports with a literature review | Internal Medicine | 2016 | Case series |
| Kunchok | Fatal reversible cerebral vasoconstriction syndrome | Journal of the Neurological Sciences | 2018 | Case series |
| Liang | Reversible cerebral vasoconstriction syndrome following red blood cells transfusion: a case series of 7 patients | Orphanet Journal Of Rare Diseases | 2015 | Case series |
| Machner | Isoflavones and gastrointestinal infection: Two potential triggers for reversible cerebral vasoconstriction syndrome | Cephalalgia | 2018 | Case series |
| Marder | Multimodal imaging of reversible cerebral vasoconstriction syndrome: a series of 6 cases | Ajnr: American Journal of Neuroradiology | 2012 | Case series |
| Murase | Isolated cortical vasogenic edema and hyperintense vessel signs may be early features of reversible cerebral vasoconstriction syndrome: Case reports | Cephalalgia | 2018 | Case series |
| Papathanasiou | Reversible cerebral vasoconstriction syndrome as a cause of thunderclap headache: a retrospective case series study | American Journal of Emergency Medicine | 2015 | Case series |
| Roongpiboonsopit | Reversible Cerebral Vasoconstriction Syndrome: A Report on Three Cases | Journal of the Medical Association of Thailand | 2016 | Case series |
| Thyden | Intravenous epoprostenol for treatment-refractory reversible cerebral vasoconstriction syndrome (RCVS) | Journal of the Neurological Sciences | 2016 | Case series |
| Wiles | Reversible cerebral vasoconstriction syndrome: a rare cause of postpartum headache | Practical Neurology | 2015 | Case series |
| Wong | Recurrent thunderclap headaches and multilobar intracerebral haemorrhages: two cases of reversible cerebral vasoconstriction syndrome (RCVS) | Cephalalgia | 2009 | Case series |
| Cho | Effect of nimodipine treatment on the clinical course of reversible cerebral vasoconstriction syndrome | Frontiers in Neurology | 2019 | Prospective observational study |
| Alons | Prediction of vascular abnormalities on CT angiography in patients with acute headache | Brain and Behaviour | 2018 | Retrospective observational study |
| Caria | The clinical spectrum of reversible cerebral vasoconstriction syndrome: The Italian project on stroke at young age | Cephalalgia | 2019 | Retrospective observational study |
| Ducros | Hemorrhagic manifestations of reversible cerebral vasoconstriction syndrome: frequency, features, and risk factors | Stroke | 2010 | Retrospective observational study |
| Fugate | Variable presentations of postpartum angiopathy | Stroke | 2012 | Retrospective observational study |
| Robert | Reversible cerebral vasoconstriction syndrome identification of prognostic factors | Clinical Neurology & Neurosurgery | 2013 | Retrospective observational study |
| Rocha | RCVS 2 score and diagnostic approach for reversible cerebral vasoconstriction syndrome | Neurology | 2019 | Retrospective observational study |

| Author Last Name | Article Title | Study Design | Quality of Score | Risk of Bias |
| --- | --- | --- | --- | --- |
| Davies | The reversible cerebral vasoconstriction syndrome in association with venlafaxine and methenamine | Case series | 14/18 | Moderate |
| Fukaguchi | Reversible cerebral vasoconstriction syndrome: the importance of follow-up imaging within 2 weeks. | Case series | 14/18 | Moderate |
| Kato | Triptan-induced reversible cerebral vasoconstriction syndrome: Two case reports with a literature review | Case series | 11/18 | High risk |
| Kunchok | Fatal reversible cerebral vasoconstriction syndrome | Case series | 10/18 | High risk |
| Liang | Reversible cerebral vasoconstriction syndrome following red blood cells transfusion: a case series of 7 patients | Case series | 10/18 | High risk |
| Machner | Isoflavones and gastrointestinal infection: Two potential triggers for reversible cerebral vasoconstriction syndrome | Case series | 9/18 | Very-high risk |
| Marder | Multimodal imaging of reversible cerebral vasoconstriction syndrome: a series of 6 cases | Case series | 9/18 | Very- high risk |
| Murase | Isolated cortical vasogenic edema and hyperintense vessel signs may be early features of reversible cerebral vasoconstriction syndrome: Case reports | Case series | 9/18 | Very-high risk |
| Papathanasiou | Reversible cerebral vasoconstriction syndrome as a cause of thunderclap headache: a retrospective case series study | Case series | 9/18 | Very-high risk |
| Roongpiboonsopit | Reversible Cerebral Vasoconstriction Syndrome: A Report on Three Cases | Case series | 9/18 | Very-high risk |
| Thyden | Intravenous epoprostenol for treatment-refractory reversible cerebral vasoconstriction syndrome (RCVS) | Case series | 9/18 | Very-high risk |
| Wiles | Reversible cerebral vasoconstriction syndrome: a rare cause of postpartum headache | Case series | 9/18 | Very-high risk |
| Wong | Recurrent thunderclap headaches and multilobar intracerebral haemorrhages: two cases of reversible cerebral vasoconstriction syndrome (RCVS) | Case series | 9/18 | Very-high risk |
| Cho | Effect of nimodipine treatment on the clinical course of reversible cerebral vasoconstriction syndrome | Prospective observational study | 9/18 | Very-high risk |
| Alons | Prediction of vascular abnormalities on CT angiography in patients with acute headache | Retrospective observational study | 8/18 | Very-high risk |
| Caria | The clinical spectrum of reversible cerebral vasoconstriction syndrome: The Italian project on stroke at young age | Retrospective observational study | 8/18 | Very-high risk |
| Ducros | Hemorrhagic manifestations of reversible cerebral vasoconstriction syndrome: frequency, features, and risk factors | Retrospective observational study | 8/9 | Low risk |
| Fugate | Variable presentations of postpartum angiopathy | Retrospective observational study | 6/9 | Low risk |
| Robert | Reversible cerebral vasoconstriction syndrome identification of prognostic factors | Retrospective observational study | 9/9 | Low risk |
| Rocha | RCVS 2 score and diagnostic approach for reversible cerebral vasoconstriction syndrome | Retrospective observational study | 8/9 | Low risk |
